# Variability in public procurement prices for Group 1A drugs in the specialized pharmaceutical component: an observational study, 2013-2022

**DOI:** 10.1590/S2237-96222025v34e20240733.en

**Published:** 2025-09-01

**Authors:** Paula Rossignoli, Roberto Pontarolo, Fernando Fernandez-Llimos

**Affiliations:** 1 Universidade Federal do Paraná, Programa de Pós-Graduação em Assistência Farmacêutica, Curitiba, PR, Brasil; 2 Universidade Federal do Paraná, Departamento de Farmácia, Curitiba, PR, Brasil; 3 Universidade do Porto, Faculdade de Farmácia, Porto, Portugal

**Keywords:** Drug Costs, Negotiating, Financing, Government, Technology, High-Cost, Observational Study, Costos de los Medicamentos, Negociación, Financiación Gubernamental, Tecnología de Alto Costo, Estudio Observacional

## Abstract

**Objective:**

To assess the difference in acquisition prices of Group 1A drugs in the Specialized Pharmaceutical Component between purchases made by the Brazilian Ministry of Health and those made by the Paraná State Health Department.

**Methods:**

This was a retrospective observational study comparing prices of medications acquired centrally by the Brazilian Ministry of Health and those acquired by the State of Paraná to meet court orders or to supply supplementary lists, from 2013 to 2022. The weighted average acquisition price was calculated for each procurement source, per year, based on data from the Paraná Health Department management information system. The ratio between Paraná’s and the Brazilian Ministry of Health’s weighted average prices was calculated, as well as the hypothetical cost of Paraná’s purchases had they been made at the Ministry’s prices. Total overpricing in Paraná’s acquisitions was also calculated.

**Results:**

A total of 500 price comparisons were conducted, covering 116 different drug presentations. In 84.6% of the comparisons, Paraná’s prices exceeded those of the Ministry of Health. For seven pharmaceutical presentations, the state price was more than 10 times higher than the federal price, reaching as high as 47.32 times in the most extreme case. Overall, Paraná’s expenditures exceeded by more than BRL 200 million the amount that would have been paid at the Ministry’s prices, resulting in 55.7% overpricing.

**Conclusion:**

There was considerable variation between prices of drugs acquired centrally by the Brazilian Ministry of Health and those acquired by the state of Paraná. To enhance cost-effectiveness within the Brazilian National Health System, mechanisms for centralized price negotiation or procurement should be further explored.

## Resumen

**Objetivo:**

Evaluar la diferencia en los precios de adquisición de medicamentos del grupo 1 del Componente Especializado de Asistencia Farmacéutica entre las compras realizadas por el Ministerio de Salud y las realizadas por la Secretaría de Estado de Salud de Paraná.

**Métodos:**

Se trata de un estudio observacional retrospectivo que compara los precios de los medicamentos adquiridos de forma centralizada por el Ministerio de Salud y los adquiridos por Paraná para demandas judiciales o lista complementaria, entre 2013 y 2022. Se calculó el precio medio ponderado de adquisición para cada una de las dos fuentes, por año, a partir de los datos del sistema de información gerencial de la secretaría de Paraná. Se calculó la relación entre el precio medio ponderado de Paraná y del Ministerio de Salud y los valores hipotéticos de las adquisiciones realizadas por Paraná si se hubieran adquirido al precio del ministerio. Se calculó el sobreprecio total de las adquisiciones en Paraná.

**Resultados:**

Se realizaron 500 comparaciones de precios de 116 presentaciones diferentes de medicamentos. En el 84,6% de los casos, el precio de Paraná superó al del Ministerio de Salud. En siete presentaciones, el precio estatal superó 10 veces al federal, llegando, en el caso más extremo, a 47,32 veces. En total, el gasto de Paraná superó en más de 200 millones de reales el valor que se habría pagado a los precios de adquisición del ministerio, lo que supuso un sobreprecio del 55,7%.

**Conclusión:**

Se observó una gran variabilidad entre los precios de las adquisiciones centralizadas por el Ministerio de Salud y las descentralizadas en Paraná. Para una mayor economía en el Sistema Único de Salud, debería profundizarse el estudio de mecanismos que centralicen la negociación de precios o la propia adquisición.

**Ethical aspects**

This research used public domain anonymized databases.

## Introduction

In health systems worldwide, so-called specialized or high-cost drugs represent one of the main components of health expenditures. Experts predict that the spread of these drugs will be one of the main factors driving growth in healthcare expenditure in the coming years (1,2). In North America, they were estimated to account for 53.0% of the total growth in drug expenditures between 2013 and 2018, while in Europe, this figure reached 94.0% over the same period. This growth stems from the increased availability of such drugs, their increasingly high prices, and the growing number of potential users (2).

In the Brazilian National Health System (*Sistema Único de Saúde* – SUS), these medicines are part of the Specialized Pharmaceutical Component and are used to treat chronic conditions, including rare diseases, as specified in the Clinical Protocols and Therapeutic Guidelines published by the Brazilian Ministry of Health. This component represents the largest share of federal expenditures on outpatient medicines (3).

For funding purposes, it is divided into two groups: Group 1, funded by the federal government, and Group 2, funded by the states. Group 1 encompasses the highest-cost drugs, including those under commercial exclusivity and those produced through product development partnerships. In terms of procurement, Group 1 is further divided into Group 1A, which is procured by the Ministry of Health, and Group 1B, which is procured at the state level (4).

Although Group 1A drugs are both funded and centrally procured by the federal government, they are often purchased by state governments under certain circumstances, such as supply shortages or delays in delivery by the Ministry of Health; or when they are used for clinical indications or under criteria that differ from those established in the SUS Clinical Protocols and Therapeutic Guidelines—whether due to expanded use authorized by state health departments or in response to court orders (5-7). The number of drugs incorporated into SUS and allocated to Group 1A has increased over the years. Of the 27 drugs incorporated into Group 1 between 2012 and 2018, 20 were assigned to Group 1A and only 7 to Group 1B. In 2015, the number of Group 1A drugs surpassed that of Group 1B (8). 

Procurement conducted in centralized scenarios may benefit from economies of scale. Comparing public procurement prices between centralized (Ministry of Health) and decentralized (state-level) purchases can demonstrate which model is more cost-effective and advantageous for public administration. At the time this study was completed, no previously published research had assessed this comparison. 

The objective of this study was to evaluate differences in prices of Group 1A drugs in the Specialized Pharmaceutical Component between purchases made by the Brazilian Ministry of Health and those made by the Paraná State Health Department from 2013 to 2022.

## Methods

This was a retrospective observational study based on data from the information system used by the Paraná State Health Department to manage the inflow, outflow, and stock of drugs, including those acquired directly by the Department and those received from the Ministry of Health. The data were provided to the authors upon authorization from the Department. Although this was an observational study, the Strengthening the Reporting of Observational Studies in Epidemiology (STROBE) initiative was not used to structure the methods, as many of its topics (e.g., participants, bias, sample size) were not applicable to this study. 

### Data collection

All records of drug receipts under the Specialized Pharmaceutical Component and those procured due to court orders between 2013 and 2022 were obtained. That dataset included the following fields: drug name, supplier, date of receipt, quantity received, and unit price. 

Given that, until the 2022 edition of the National List of Essential Drugs, the medicines were not identified by the different groups of the Specialized Pharmaceutical Component to which they belonged, all those that were ever supplied by the Ministry of Health during the analysis period were identified as potentially belonging to group 1A. For each of these drugs, we checked the Management System for the Table of Procedures, Drugs, Orthotics, Prosthetics, and Special Materials of the Brazilian National Health System to verify the year of inclusion in the specialized component, linkage to group 1A, possible migration between group 1A and group 1B, or exclusion from the component (Supplementary Table 1) (9). 

**Table 1:** Number of pharmaceutical presentations, units, and values of Group 1A drugs in the Specialized Pharmaceutical Component purchased by the Brazilian Ministry of Health and the Paraná State Health Department, 2013-2022

	Brazilian Ministry of Health			Paraná State Health Department		
Year	Pharmaceutical presentations	Units	Nominal value (BRL)	Pharmaceutical presentations	Units	Nominal value (BRL)
2013	61	21,267,772	196,111,934.08	27	384,441	15,396,918.22
2014	64	18,444,825	210,214,082.80	31	2,768,294	18,873,512.81
2015	70	25,320,270	248,455,118.84	43	1,250,209	34,743,421.04
2016	67	27,021,851	294,549,940.29	43	1,573,380	37,234,875.79
2017	74	30,486,624	266,740,540.42	46	1,329,577	30,721,475.05
2018	80	34,302,931	247,745,716.00	66	4,685,530	43,241,884.72
2019	110	48,198,953	328,687,042.58	66	3,920,230	66,078,753.16
2020	122	48,798,501	334,229,336.51	70	3,478,411	67,908,031.47
2021	126	55,326,298	400,434,217.12	64	2,074,132	37,219,630.82
2022	129	58,919,394	384,287,060.30	67	2,903,115	33,055,948.59
Total		368,087,419	2,911,454,988.95		24,367,319	384,474,451.67

For each drug, records from the imported information system file that were outside the period of association of the drug with group 1A were excluded. The final file identified entries that came from the Ministry of Health, with the remaining entries corresponding to purchases made by the Paraná State Health Department, either for the supplementary list or to comply with court orders.

### Data analysis

For each procurement, the total acquisition cost was calculated by multiplying the unit price by the quantity received.


$$V_{t}=P_{u}*Q$$

The weighted average acquisition price was calculated for each procurement source and year as the quotient of the total value of purchases from that source in that year divided by the total number of units procured. 


$$PMP=\frac{\sum V_{t_{i}}}{\sum Q_{i}\;}$$

For each year, the ratio between Paraná’s weighted average price (PMP) and that of the Brazilian Ministry of Health (MS) was calculated. Values above 1 indicate that the price paid by the Paraná State Health Department (SES) was higher than that of the Ministry of Health.

$$PMP\left(\frac{SES}{MS}\right)=\frac{PMP_{SES}}{PMP_{MS}}$$

For each year, hypothetical values were calculated for the procurement of each drug by the Paraná State Health Department if they had been purchased at the weighted average price of the procurements received from the Ministry of Health for that drug in that year.

$$V_{h_{SES}}=PMP_{MS}*Q_{SES}$$

To estimate the overpricing or cost savings resulting from decentralized procurement, the difference was calculated between the total value of purchases of each medicine by the Paraná State Health Department and the corresponding hypothetical values of these purchases if they had been centralized.

$$\mathrm{Overpricing}=V_{real_{SES}}-V_{h_{SES}}$$

All acquisition prices were adjusted to 2022 levels using the annual variation in the national broad consumer price index calculated by the Brazilian Institute of Geography and Statistics (IBGE) (10). The calculation period was established from June 2013 to June 2022.

## Results

Between 2013 and 2022, the Paraná State Health Department received 392,454,738 units of drugs belonging to the Specialized Pharmaceutical Component and also due to court orders. Of this total, 368,087,419 units, corresponding to 151 different pharmaceutical presentations, were drugs procured by the Brazilian Ministry of Health (group 1A). Of these 151 presentations, 120 were also acquired by Paraná State Health Department during the same period, resulting in 2,449 procurement records. The total amount spent by the federal government was BRL 2,911,454,988.95 (nominal value), equivalent to BRL 3,792,571,464.52 when adjusted to 2022 values. The total amount spent by the State of Paraná was BRL 384,474,451.67 (nominal value), equivalent to BRL 498,191,292.43 (2022-adjusted value) (Table 1). 

Of the 120 pharmaceutical presentations of Group 1A drugs with both centralized and decentralized procurement, 116 presentations, corresponding to 77 active ingredients, were procured in the same years, which enabled a paired comparison of purchase prices in the same year, totaling 500 comparisons. In 423 comparisons (84.6%), the price paid by Paraná exceeded that of the Ministry of Health; in 76 (15.2%), the opposite occurred; and in one case, both institutions paid the same amount. In 42 pharmaceutical presentations, the state procurement exceeded the federal one in five or more years of the analysis, reaching six presentations in which the price paid by the Department was higher than that paid by the Ministry of Health in all 10 years of the analysis. 

In seven pharmaceutical presentations, the overpricing for Paraná exceeded 10 times the Ministry of Health price, with the most extreme case being ribavirin 250 mg, which in 2016 was purchased by the Department at a price 47.32 times higher than the centralized procurement price. Conversely, the procurement price paid by the state was lower in some cases, such as for quetiapine 200 mg, with a state-to-federal price ratio of 0.21 in 2015.

For most pharmaceutical presentations, price differences remained relatively stable over the 10-year period. However, two presentations showed substantial increases in price disparity: adalimumab 40 mg, with a price ratio increasing from 1.83 in 2013 to 18.35 in 2022; and cinacalcet 30 mg, from 1.38 in 2017 to 19.63 in 2022 (9).

Considering the 116 pharmaceutical presentations eligible for paired analysis, the total expenditure by the Paraná State Health Department was BRL 370,857,537.87 (nominal value), or BRL 480,865,617.89 (adjusted to 2022). This amount exceeded by BRL 206,666,073.79 (nominal value), or BRL 267,370,342.38 (2022-adjusted), what would have been paid had the purchases been made at federal prices in the same years, resulting in an overpricing rate of 55.7% (Table 2). Across the 10-year study period, the 14 presentations for which Paraná paid less than the Brazilian Ministry of Health resulted in a savings of BRL 1,652,991.24. The remaining 102 pharmaceutical presentations accounted for an overpricing of BRL 208,319,065.03, leading to a total overpricing of BRL 206,666,073.79, or BRL 267,370,342.40 when adjusted to 2022 values (9). 

**Table 2:** Procurement values of Group 1A drugs in the Specialized Pharmaceutical Component by the Paraná State Health Department and comparison with purchases by the Brazilian Ministry of Health, 2013-2022

Year	Paraná’s nominal value (BRL)	Paraná’s most expensive pharmaceutical presentations	Paraná’s cheapest pharmaceutical presentations	Paraná’s Overpricing	Overpricing (%)
2013	15,396,918.22	21	6	6,082,158.64	39.5
2014	18,873,445.17	23	7	8,355,783.80	44.3
2015	34,743,326.22	33	9	21,059,848.52	60.6
2016	36,922,264.00	34	6	22,687,323.44	61.4
2017	30,721,363.48	40	5	21,170,321.46	68.9
2018	31,884,346.08	46	8	20,171,140.62	63.3
2019	66,057,971.71	57	8	37,679,606.80	57.0
2020	67,100,801.18	56	11	31,639,135.66	47.2
2021	37,219,630.82	56	8	20,440,001.40	54.9
2022	31,937,470.99	57	8	17,380,753.44	54.4
Total	370,857,537.87			206,666,073.79	55.7

The drug with the largest absolute price difference between institutions was nusinersen, purchased in 2022 at BRL 159,999.40 by the Ministry of Health and BRL 280,281.45 by Paraná. Given the quantity procured by the state, this resulted in BRL 13.2 million overpricing (9). The highest cumulative overpricing values during the 10-year period were observed for adalimumab 40 mg and mycophenolate mofetil, totaling BRL 31.0 million and BRL 30.6 million, respectively (9).

## Discussion

The analysis of this 10-year longitudinal study with actual procurement data revealed significant disparities in the prices of Group 1A drugs. It was found that centralized procurement by the Brazilian Ministry of Health was, in the vast majority of cases, was at a much lower price than decentralized procurement by the Paraná State Health Department. The total cost of Group 1A drug purchases made by the state entity over the 10-year period analyzed was BRL 200 million higher than if they had been purchased at the Ministry of Health price, which would correspond to BRL 267 million in 2022. 

Paraná was used as a case study due to its robust information system that aggregates data on drugs purchased and supplied by the Ministry of Health and those purchased by the Paraná State Health Department. In a study on public procurement prices for Group 1B drugs in the Specialized Pharmaceutical Component by state health departments, Paraná was among the states that obtained comparatively lower procurement prices (11). Considering that the state represents only 5.6% of Brazil’s population, if the price differences obtained in this study were extrapolated to the other federal units in the country, total overpricing would be more than BRL 4.7 billion at 2022 values.

A limitation of the study is the possible underestimation of the total overpricing of decentralized purchases, since, to be more reliable, comparisons were only made for purchases made by the two entities in the same year. The study was conducted only in Paraná, and although there is no reason to believe that there may be significant differences with other states, the data should be validated before generalization.

Among the main reasons for the procurement of Group 1A drugs by state health departments are court orders that require them to provide these drugs for clinical conditions or criteria other than those provided for in the Clinical Protocols and Therapeutic Guidelines of the Brazilian National Health System (12,13). The judicialization of health, especially for drugs, is a frequent phenomenon in Latin American countries (14-16). Judicialization leads to the creation of policies parallel to the Brazilian National Health System, which disrupts and compromises public drug policy, widening inequalities among users (17,18). Despite intense debates and studies on the judicialization of health, the numbers have not pointed to a reduction in litigation (19,20). Lawsuits for the provision of medicines have been identified as a mechanism used by the pharmaceutical industry to introduce new medicines into the Brazilian National Health System (7,21).

A second reason for the decentralized procurement of Group 1A drugs was the existence of a complementary list maintained by state health departments. In general, this list corresponds to the extension of use for clinical situations or criteria not covered by the Clinical Protocols and Therapeutic Guidelines of the Brazilian National Health System. States and municipalities may have complementary lists of drugs, as provided for by Federal Decree No. 7508/2011. This practice, however, may lead to inequalities in access to drugs in the Brazilian National Health System between states, the Federal District, and municipalities, favoring users residing in states or municipalities with greater wealth and budgetary resources (22,23).

Two situations influenced the total overpricing paid by the Paraná State Health Department: i) a large difference in procurement prices between the Brazilian Ministry of Health and the Department; and ii) the large quantity purchased by the Department. Nusinersen, an example of the first situation, was incorporated into the Brazilian National Health System in 2019 for the treatment of spinal muscular atrophy type 1 (24), and presented a price difference between federal and state-level procurement. The unit price ranged from BRL 91,400 to BRL 120,000 per vial in purchases made between 2019 and 2022. 

Adalimumab 40 mg accounted for the highest accumulated overpricing due to the large volume acquired by the state. The price difference between the Ministry of Health and the Paraná State Health Department increased over time, from 1.83 times higher in 2013 to 18.35 times higher in 2022. This difference became more pronounced in the last three years of the analysis, during which the price paid by the state remained unchanged, while a substantial decrease was observed in the price paid by the Ministry of Health, likely due to a Productive Development Partnership (PDP) established during the period (25). However, a similar trend was observed for cinacalcet, despite the absence of any such partnership.

Conversely, some drugs, such as quetiapine 25 mg, 100 mg, and 200 mg, were acquired by Paraná at lower prices than those paid by the Brazilian Ministry of Health, even though they were also under a PDP. It appears that in this case, the contract price established in the agreement may have been higher than the price subsequently charged by the market. Although these partnerships should be associated with price reductions, studies have shown that there are exceptions (26,27). 

The findings of this study indicate that, in most cases, centralized procurement of drugs results in greater cost-efficiency. The advantages of centralized procurement policies have also been observed for group 1B drugs under the Specialized Pharmaceutical Component (11). Centralization may refer either to the procurement process itself, conducted by the Brazilian Ministry of Health, or to centralized price negotiation by the Ministry for acquisitions carried out by state health departments. Centralized price negotiation with fixed procurement prices in public health systems is a strategy adopted by many countries (28-30). Either of these approaches is expected to reduce procurement costs. This appears to be the model proposed for oncology drugs incorporated into SUS following the enactment of Law No. 14758/2023, which encompasses both options. 

To enhance cost-efficiency within SUS, further studies on mechanisms to centralize price negotiation or expand centralized procurement are warranted.

## Data Availability

The detailed results were made available in the Open Science Framework under the digital object identifier: 10.17605/OSF.IO/CS8MW. The original data used for the calculations are the property of the Paraná State Health Department and should be provided by them upon justified request.
