# Supplementary material for: Variability in public procurement prices for Group 1A drugs in the specialized pharmaceutical component: an observational study, 2013-2022
Source: Epidemiol Serv Saude. 2025 Sep 1;34:e20240733. doi: 10.1590/S2237-96222025v34e20240733.en (PMC12404603; doi:10.1590/S2237-96222025v34e20240733.en)
Supplement: Translation

# Translation: Variabilidade nos preços de aquisição pública de medicamentos do grupo 1A do componente especializado da assistência farmacêutica: estudo observacional, 2013-2022

**DOI:** 10.1590/S2237-96222025v34e20240733.pt

**Authors:** Paula Rossignoli, Roberto Pontarolo, Fernando Fernandez-Llimos

**Affiliations:** 1 Universidade Federal do Paraná, Programa de Pós-Graduação em Assistência Farmacêutica, Curitiba, PR, Brasil; 2 Universidade Federal do Paraná, Departamento de Farmácia, Curitiba, PR, Brasil; 3 Universidade do Porto, Faculdade de Farmácia, Porto, Portugal

**Keywords:** Custos de Medicamentos, Negociação, Financiamento Governamental, Tecnologia de Alto Custo, Estudo Observacional

## Abstract

**Objetivo:**

Avaliar a diferença nos preços de aquisição de medicamentos do grupo 1A do Componente Especializado da Assistência Farmacêutica entre as compras realizadas pelo Ministério da Saúde e as realizadas pela Secretaria de Estado da Saúde do Paraná.

**Métodos:**

Tratou-se de estudo observacional retrospectivo comparando preços de medicamentos adquiridos centralizadamente pelo Ministério da Saúde e adquiridos pelo Paraná para demandas judiciais ou elenco complementar, entre 2013 e 2022. O preço médio ponderado de aquisição foi calculado para cada uma das duas origens, por ano, a partir dos dados do sistema de informação gerencial da secretaria do Paraná. Calcularam-se a razão entre o preço médio ponderado do Paraná e do Ministério da Saúde e os valores hipotéticos das aquisições realizadas pelo Paraná caso tivessem sido adquiridas pelo preço do ministério. Foi calculado o sobrepreço total das aquisições no Paraná.

**Resultados:**

Realizaram-se 500 comparações de preços de 116 diferentes apresentações de medicamentos. Em 84,6%, o preço do Paraná superou o do Ministério da Saúde. Em sete apresentações, o preço estadual superou 10 vezes o federal, chegando, no caso mais extremo, a 47,32 vezes. No total, o gasto do Paraná excedeu em mais de R$ 200 milhões o valor que seria pago aos preços de aquisição do ministério, resultando no sobrepreço de 55,7%.

**Conclusão:**

Verificou-se grande variabilidade entre preços de aquisições centralizadas pelo Ministério da Saúde e descentralizadas no Paraná. Para maior economicidade no Sistema Único de Saúde, o estudo de mecanismos que centralizem a negociação de preços ou a própria aquisição deveria ser aprofundado.

**Aspectos éticos**

Esta pesquisa utilizou bancos de dados de domínio público e anonimizados.:

## Introdução

Nos sistemas de saúde em todo o mundo, os medicamentos denominados especializados ou de alto custo constituem um dos principais elementos do gasto em saúde. A projeção de especialistas é de que a propagação desses medicamentos será um dos principais impulsionadores do crescimento dos gastos com saúde nos próximos anos (1,2). Na América do Norte, estimou-se que eles representariam 53,0% do crescimento total de gastos com medicamentos entre 2013 e 2018, enquanto na Europa seria 94,0% no mesmo período. Esse crescimento decorre da maior disponibilidade desses medicamentos, dos preços cada vez mais elevados e do número crescente de usuários potenciais (2).

No Sistema Único de Saúde, esses fármacos estão inseridos no Componente Especializado da Assistência Farmacêutica e são utilizados para tratar enfermidades crônicas, incluindo condições raras, conforme especificado nos Protocolos Clínicos e Diretrizes Terapêuticas publicados pelo Ministério da Saúde. Esse componente representa a maior parcela do dispêndio federal com medicamentos em nível ambulatorial (3).

Para fins de financiamento, ele é segmentado em dois grupos: grupo 1, custeado pelo ente federal, e grupo 2, custeado pelos estados. O grupo 1 abrange os medicamentos de maior valor financeiro, incluindo aqueles sob exclusividade comercial e os produzidos por meio de acordos de desenvolvimento produtivo. No que diz respeito à aquisição, ele se subdivide em grupos 1A, com compra pelo Ministério da Saúde, e 1B, com aquisição descentralizada nos estados (4).

Embora sejam de financiamento e de aquisição centralizados pelo gestor federal, os medicamentos que compõem o grupo 1A são muitas vezes adquiridos de forma descentralizada pelos estados em casos como: desabastecimento ou atraso no envio pelo Ministério da Saúde; e utilização para condições clínicas ou com critérios diferentes daqueles previstos nos Protocolos Clínicos e Diretrizes Terapêuticas do Sistema Único de Saúde, quer seja em função de ampliação de uso pelas secretarias estaduais de saúde, quer seja para o cumprimento de ações judiciais (5-7). O número de medicamentos incorporados ao Sistema Único de Saúde e alocados no grupo 1A vem crescendo ao longo dos anos. Dos 27 medicamentos incorporados e alocados no grupo 1 entre 2012 e 2018, 20 foram alocados no grupo 1A, e apenas 7, no grupo 1B. Em 2015, o número de medicamentos do grupo 1A superou o do grupo 1B (8). 

Os processos de compra realizados em cenários centralizados podem usufruir de benefícios associados às economias de escala. Comparar os preços de aquisição pública em compras centralizadas (realizadas pelo Ministério da Saúde) e descentralizadas (nos estados) poderá demonstrar o modelo de maior economicidade e vantajosidade para a administração pública. Até a conclusão desta pesquisa, não havia sido publicado estudo que tenha realizado essa avaliação. 

O objetivo desta pesquisa foi avaliar a diferença nos preços de medicamentos do grupo 1A do Componente Especializado da Assistência Farmacêutica entre as compras realizadas pelo Ministério da Saúde e as realizadas pela Secretaria de Estado da Saúde do Paraná entre 2013 e 2022.

## Métodos

Tratou-se de estudo observacional retrospectivo com dados de sistema de informação próprio da Secretaria de Estado da Saúde do Paraná, utilizado para a gestão de entradas, saídas e estoque de medicamentos, incluindo os adquiridos pela própria secretaria e aqueles recebidos do Ministério da Saúde. Os dados foram fornecidos aos autores após a autorização da secretaria. Apesar de ser um estudo observacional, não foi utilizada a iniciativa Strengthening the Reporting of Observational Studies in Epidemiology (STROBE) para estruturar os métodos, porque muitos dos tópicos previstos não seriam aplicados neste estudo, a exemplo de participantes, viés e tamanho do estudo. 

### Coleta de dados

Foram obtidos todos os registros de recebimento de medicamentos que compunham o Componente Especializado da Assistência Farmacêutica e de medicamentos adquiridos para demanda judicial entre 2013 e 2022. Esse arquivo estava composto pelos seguintes campos: denominação do medicamento, fornecedor, data de entrada, quantidade recebida e valor unitário. 

Dado que, até a edição de 2022 da Relação Nacional de Medicamentos Essenciais, os medicamentos não eram identificados pelos diferentes grupos do Componente Especializado da Assistência Farmacêutica ao que pertenciam, foram identificados como medicamentos potencialmente do grupo 1A todos aqueles que foram fornecidos alguma vez pelo Ministério da Saúde no período de análise. Para cada um desses medicamentos, foi realizada a conferência no Sistema de Gerenciamento da Tabela de Procedimentos, Medicamentos e Órteses, Próteses e Materiais Especiais do Sistema Único de Saúde para verificação do ano de inclusão no componente especializado, vinculação ao grupo 1A, eventual migração entre grupo 1A e grupo 1B, ou exclusão do componente (Tabela suplementar 1) (9). 

Para cada um dos medicamentos, foram excluídos os registros do arquivo importado do sistema de informação que estavam fora do período de vinculação do medicamento ao grupo 1A. Nesse arquivo final, foram identificadas as entradas que vieram do Ministério da Saúde, sendo as demais entradas correspondentes a aquisições realizadas pela Secretaria de Estado da Saúde do Paraná, quer seja para o elenco complementar, quer seja para o cumprimento de demandas judiciais.

### Análise de dados

Para cada aquisição, foi calculado o valor total da aquisição pelo produto entre o valor unitário e a quantidade recebida.


$$V_{t}=P_{u}*Q$$

O preço médio ponderado de aquisição foi calculado para cada uma das duas origens em cada ano como o quociente entre o somatório de valores totais de aquisição obtidos dessa origem nesse ano e o somatório do número de unidades dessas aquisições. 


$$PMP=\frac{\sum V_{t_{i}}}{\sum Q_{i}\;}$$

Para cada ano, foi calculada a razão entre o preço médio ponderado de aquisição pela Secretaria de Estado da Saúde do Paraná e o preço médio ponderado da aquisição pelo Ministério da Saúde. Valores acima de 1 indicam que o preço pago pela secretaria foi maior do que o do Ministério da Saúde. 


$$PMP\left(\frac{SES}{MS}\right)=\frac{PMP_{SES}}{PMP_{MS}}$$

Para cada ano, foram calculados os valores hipotéticos das aquisições de cada medicamento realizadas pela Secretaria de Estado da Saúde do Paraná caso tivessem sido adquiridas pelo preço médio ponderado das aquisições recebidas do Ministério da Saúde nesse ano desse medicamento. 


$$V_{h_{SES}}=PMP_{MS}*Q_{SES}$$

Para estimar o sobrepreço ou a poupança implicados pela aquisição descentralizada, foi calculada a diferença entre o valor total das aquisições da Secretaria de Estado da Saúde do Paraná de cada medicamento e os correspondentes valores hipotéticos dessas aquisições caso tivessem sido centralizadas. 


$$\mathrm{Sobrepreço}=V_{real_{SES}}-V_{h_{SES}}$$

Todos os preços de aquisição foram corrigidos para 2022, empregando-se a variação anual do índice nacional de preços ao consumidor amplo por meio da calculadora do Instituto Brasileiro de Geografia e Estatística (10). O período de cálculo foi estabelecido de junho do ano em questão a junho de 2022.

## Resultados

Entre 2013 e 2022, a Secretaria de Estado da Saúde do Paraná recebeu 392.454.738 unidades de medicamentos do Componente Especializado da Assistência Farmacêutica e de demanda judicial. Deste total, 368.087.419 unidades, correspondendo a 151 diferentes apresentações, eram de medicamentos adquiridos pelo Ministério da Saúde (grupo 1A). Destas 151, 120 também haviam sido adquiridas pela secretaria ao longo do período, resultando em 2.449 registros de entrada. O valor total das aquisições realizadas pelo ente federal foi R$ 2.911.454.988,95 (valor nominal) correspondente a R$ 3.792.571.464,52 (valor atualizado para 2022). O valor total das aquisições realizadas pelo Paraná foi R$ 384.474.451,67 (valor nominal) correspondente a R$ 498.191.292,43 (valor atualizado para 2022) (Tabela 1). 

**Tabela 1:** Número de apresentações, unidades e valores de medicamentos do grupo 1A do componente especializado da assistência farmacêutica adquiridos pelo Ministério da Saúde e pela Secretaria de Estado da Saúde do Paraná, 2013-2022

	Ministério da Saúde			Secretaria de Estado da Saúde do Paraná		
Ano	Apresentações	Unidades	Valor nominal (R$)	Apresentações	Unidades	Valor nominal (R$)
2013	61	21.267.772	196.111.934,08	27	384.441	15.396.918,22
2014	64	18.444.825	210.214.082,80	31	2.768.294	18.873.512,81
2015	70	25.320.270	248.455.118,84	43	1.250.209	34.743.421,04
2016	67	27.021.851	294.549.940,29	43	1.573.380	37.234.875,79
2017	74	30.486.624	266.740.540,42	46	1.329.577	30.721.475,05
2018	80	34.302.931	247.745.716,00	66	4.685.530	43.241.884,72
2019	110	48.198.953	328.687.042,58	66	3.920.230	66.078.753,16
2020	122	48.798.501	334.229.336,51	70	3.478.411	67.908.031,47
2021	126	55.326.298	400.434.217,12	64	2.074.132	37.219.630,82
2022	129	58.919.394	384.287.060,30	67	2.903.115	33.055.948,59
Total		368.087.419	2.911.454.988,95		24.367.319	384.474.451,67

Das 120 apresentações de medicamentos do grupo 1A com aquisições tanto centralizadas quanto descentralizadas, 116 apresentações, correspondentes a 77 princípios ativos, tiveram aquisições em anos coincidentes, o que possibilitou a comparação emparelhada de preços de compra no mesmo ano, totalizando 500 comparações. Em 423 (84,6%) destas, o preço do Paraná superou o das aquisições centralizadas do ministério; em 76 (15,2%) ocorreu o inverso; e em uma apresentação o preço de aquisição foi o mesmo para as duas instituições. Em 42 apresentações, o preço do ente estadual superou o do federal em cinco ou mais anos da análise, chegando-se a seis apresentações em que o preço da secretaria foi superior ao do Ministério da Saúde em todos os 10 anos de análise. 

Em sete apresentações, o sobrepreço para o Paraná superou 10 vezes o preço do Ministério da Saúde, sendo o caso mais extremo o da ribavirina 250 mg que, em 2016, teve preço de aquisição na secretaria 47,32 vezes o preço da aquisição centralizada. Por outro lado, o preço de aquisição pelo estado foi inferior em alguns casos, como a quetiapina 200 mg, com razão de preço estadual sobre federal de 0,21 em 2015.

Foi possível verificar que, para a maior parte das apresentações, a diferença de preços se manteve bastante constante durante os 10 anos. No entanto, destacam-se duas apresentações com incrementos importantes na diferença de preço ao longo dos anos: o adalimumabe 40 mg, que passou de uma razão de 1,83 vez em 2013 para 18,35 vezes em 2022, e o cinacalcete 30 mg, que passou de 1,38 vez em 2017 para 19,63 vezes em 2022 (9).

Considerando as 116 apresentações em que foi possível a análise emparelhada, o valor total gasto pela Secretaria de Estado da Saúde do Paraná foi R$ 370.857.537,87 (valor nominal), correspondente a R$ 480.865.617,89 (valor atualizado para 2022), superando em R$ 206.666.073,79 (valor nominal), equivalente a R$ 267.370.342,38 (valor atualizado para 2022), o valor dessas aquisições, caso tivessem sido realizadas pelos preços pagos pelo Ministério da Saúde nos mesmos anos, o que resultou no sobrepreço de 55,7% (Tabela 2). Nos 10 anos de estudo, as 14 apresentações em que o preço do Paraná foi inferior ao do Ministério da Saúde resultaram na poupança de R$ 1.652.991,24. As 102 apresentações em que ocorreram o inverso resultaram no sobrepreço de R$ 208.319.065,03, chegando-se ao valor de sobrepreço total para o ente estadual de R$ 206.666.073,79 correspondente a R$ 267.370.342,40 em valor atualizado em 2022 (9). 

**Tabela 2:** Valores de aquisição de medicamentos do grupo 1A do componente especializado da assistência farmacêutica pela Secretaria de Estado da Saúde do Paraná e comparação com aquisições do Ministério da Saúde, 2013-2022

Ano	Valor nominal aquisição (Paraná (R$))	Apresentações mais caras (Paraná)	Apresentações mais baratas (Paraná)	Sobrepreço (Paraná)	Sobrepeso (%)
2013	15.396.918,22	21	6	6.082.158,64	39,5
2014	18.873.445,17	23	7	8.355.783,80	44,3
2015	34.743.326,22	33	9	21.059.848,52	60,6
2016	36.922.264,00	34	6	22.687.323,44	61,4
2017	30.721.363,48	40	5	21.170.321,46	68,9
2018	31.884.346,08	46	8	20.171.140,62	63,3
2019	66.057.971,71	57	8	37.679.606,80	57,0
2020	67.100.801,18	56	11	31.639.135,66	47,2
2021	37.219.630,82	56	8	20.440.001,40	54,9
2022	31.937.470,99	57	8	17.380.753,44	54,4
Total	370.857.537,87			206.666.073,79	55,7

O medicamento com maior diferença de preço entre as duas instituições foi o nusinersena, adquirido em 2022 por R$ 159.999,40 pelo Ministério da Saúde e por R$ 280.281,45 pelo Paraná. Dado o número de unidades adquiridas pelo estado, o sobrepreço desse medicamento totalizou R$ 13,2 milhões (9). Os maiores valores de sobrepreço acumulados durante os 10 anos de análise corresponderam ao adalimumabe 40 mg e ao micofenolato de mofetila, o que totalizou R$ 31,0 milhões e R$ 30,6 milhões (9).

## Discussão

As análises deste estudo longitudinal de 10 anos com dados reais de aquisição revelaram disparidade significativa nos preços dos medicamentos do grupo 1A. Evidenciou-se que as aquisições centralizadas pelo Ministério da Saúde apresentaram, na grande maioria dos casos, valores muito mais baixos em comparação com as aquisições descentralizadas na Secretaria de Estado da Saúde do Paraná. O conjunto de aquisições de medicamentos do grupo 1A, realizadas pelo ente estadual nos 10 anos de análise, custou R$ 200 milhões a mais do que se tivessem sido adquiridas ao preço do Ministério da Saúde, o que corresponderia a R$ 267 milhões em valor atualizado para 2022. 

O Paraná foi utilizado como estudo de caso por dispor de sistema de informação robusto que agrega dados dos medicamentos adquiridos e enviados pelo Ministério da Saúde e aqueles adquiridos pela Secretaria de Estado da Saúde do Paraná. Em estudo sobre preços de aquisição pública de medicamentos do grupo 1B do Componente Especializado da Assistência Farmacêutica pelas secretarias estaduais de saúde, o Paraná esteve entre os estados que obtiveram, comparativamente, preços mais baixos de aquisição (11). Considerando que o estado representa apenas 5,6% da população do Brasil, se fossem extrapoladas as diferenças de preço obtidas neste estudo para as demais unidades federativas do país, o sobrepreço total seria de mais de R$ 4,7 bilhões em valor atualizado para 2022.

Como limitação do estudo, está a possível subestimação do valor total de sobrepreço das aquisições descentralizadas, uma vez que, para serem mais confiáveis, só foram realizadas comparações quando houve aquisições das duas entidades no mesmo ano. O estudo foi realizado apenas no Paraná e, ainda que nada leve a pensar que possa haver grandes diferenças com outros estados, os dados deveriam ser validados antes da generalização.

Entre os principais motivos para a aquisição de medicamentos do grupo 1A pelas secretarias estaduais de saúde, estão as demandas judiciais que obrigam as secretarias a fornecerem esses medicamentos para condições clínicas ou critérios diversos daqueles previstos nos Protocolos Clínicos e Diretrizes Terapêuticas do Sistema Único de Saúde (12,13). A judicialização da saúde, especialmente para medicamentos, é fenômeno frequente em países da América Latina (14-16). A judicialização leva à criação de política paralela ao Sistema Único de Saúde, que desorganiza e compromete a política pública de medicamentos, ampliando as desigualdades entre os usuários (17,18). Apesar de intensos debates e estudos sobre a judicialização da saúde, os números não apontaram para reduções dos litígios (19,20). As ações judiciais para fornecimento de medicamentos foram apontadas como mecanismo da indústria farmacêutica para a introdução de novos medicamentos no Sistema Único de Saúde (7,21).

Um segundo motivo para a aquisição descentralizada de medicamentos do grupo 1A foi a existência de elenco complementar das secretarias estaduais de saúde. Em geral, corresponde à extensão de uso para situações clínicas ou critérios não contemplados nos Protocolos Clínicos e Diretrizes Terapêuticas do Sistema Único de Saúde. Estados e municípios podem dispor de listas complementares de medicamentos, conforme previsto no Decreto Federal nº 7.508/2011. Essa prática, no entanto, pode levar a desigualdades no acesso a medicamentos no Sistema Único de Saúde entre estados, Distrito Federal e municípios, privilegiando aqueles usuários residentes em estados ou municípios com maior riqueza e lastro orçamentário (22,23).

Houve duas situações que influenciaram o valor total do sobrepreço pago pela Secretaria de Estado da Saúde do Paraná: i) grande diferença de preço de aquisição entre o Ministério da Saúde e a secretaria; e ii) grande quantidade adquirida pela secretaria. O medicamento nusinersena, exemplo do primeiro caso, foi incorporado ao Sistema Único de Saúde em 2019 para atrofia muscular espinhal tipo 1 (24) e teve diferença de preço entre aquisição do ente federal e do ente estadual. O preço oscilou de R$ 91,4 mil a R$ 120 mil por frasco nas aquisições entre 2019 e 2022. 

O adalimumabe 40 mg teve o maior valor acumulado de sobrepreço em decorrência de grande quantidade adquirida. A diferença de preço entre Ministério da Saúde e Secretaria de Estado da Saúde do Paraná cresceu ao longo dos anos, de 1,83 vez em 2013 até 18,35 vezes em 2022. Essa diferença se acentuou nos três últimos anos da análise, nos quais não houve variação de preço para a secretaria e, sim, importante diminuição do preço para o Ministério da Saúde, provavelmente resultante de Parceria para o Desenvolvimento Produtivo estabelecida (25). No entanto, um perfil semelhante ocorreu com cinacalcete sem que existisse nesse caso qualquer parceria estabelecida.

Situação oposta aconteceu com alguns medicamentos, como quetiapina 25 mg, 100 mg e 200 mg, que tiveram preço de aquisição pela secretaria inferior ao do ministério, apesar de também estar sob Parceria para o Desenvolvimento Produtivo. Parece que nesse caso o preço de contratação estabelecido no acordo pode ter ficado acima daquele praticado posteriormente pelo mercado. Embora essas parcerias devessem estar associadas à redução de preços, estudos demonstraram que há exceções (26,27). 

Os achados deste estudo apontam que, na maioria dos casos, a centralização da aquisição de medicamentos leva à economicidade. O benefício dessa política centralizada também foi apontado para os medicamentos do grupo 1B do Componente Especializado da Assistência Farmacêutica (11). A centralização pode ser com relação à aquisição propriamente dita, realizada pelo Ministério da Saúde, ou em relação à negociação de preços centralizada pelo Ministério da Saúde para as aquisições realizadas pelas secretarias estaduais de saúde. A negociação centralizada com fixação de preços de aquisição em sistemas públicos é adotada em muitos países (28-30). Qualquer uma dessas duas opções levaria à redução de preço de aquisição. Esse parece ser o novo modelo previsto para medicamentos oncológicos incorporados ao Sistema Único de Saúde com a publicação da Lei nº 14.758/2023, que contempla as duas opções. 

Para maior economicidade no Sistema Único de Saúde, o estudo de mecanismos que centralizem a negociação de preços ou a expansão das aquisições centralizadas deveria ser aprofundado.

## Data Availability

Os resultados pormenorizados foram disponibilizados em Estrutura de Ciência Aberta no identificador de objeto digital: 10.17605/OSF.IO/CS8MW. Os dados originais, utilizados para os cálculos, são de propriedade da Secretaria de Estado da Saúde do Paraná, devendo ser fornecido por eles mediante pedido justificado.
